# Patterns of multisite pain and associations with risk factors

**DOI:** 10.1016/j.pain.2013.05.039

**Published:** 2013-09

**Authors:** David Coggon, Georgia Ntani, Keith T. Palmer, Vanda E. Felli, Raul Harari, Lope H. Barrero, Sarah A. Felknor, David Gimeno, Anna Cattrell, Sergio Vargas-Prada, Matteo Bonzini, Eleni Solidaki, Eda Merisalu, Rima R. Habib, Farideh Sadeghian, M. Masood Kadir, Sudath S.P. Warnakulasuriya, Ko Matsudaira, Busisiwe Nyantumbu, Malcolm R. Sim, Helen Harcombe, Ken Cox, Maria H. Marziale, Leila M. Sarquis, Florencia Harari, Rocio Freire, Natalia Harari, Magda V. Monroy, Leonardo A. Quintana, Marianela Rojas, Eduardo J. Salazar Vega, E. Clare Harris, Consol Serra, J. Miguel Martinez, George Delclos, Fernando G. Benavides, Michele Carugno, Marco M. Ferrario, Angela C. Pesatori, Leda Chatzi, Panos Bitsios, Manolis Kogevinas, Kristel Oha, Tuuli Sirk, Ali Sadeghian, Roshini J. Peiris-John, Nalini Sathiakumar, A. Rajitha Wickremasinghe, Noriko Yoshimura, Helen L. Kelsall, Victor C.W Hoe, Donna M. Urquhart, Sarah Derrett, David McBride, Peter Herbison, Andrew Gray

**Affiliations:** a Medical Research Council Lifecourse Epidemiology Unit, University of Southampton, Southampton, UK; b School of Nursing, University of São Paulo, São Paulo, Brazil; c Corporación para el Desarrollo de la Producción y el Medio Ambiente Laboral–IFA (Institute for the Development of Production and the Work Environment), Quito, Ecuador; d Department of Industrial Engineering, School of Engineering, Pontificia Universidad Javeriana, Bogotá, Colombia; e Southwest Center for Occupational and Environmental Health, The University of Texas Health Science Center at Houston School of Public Health, Houston, TX, USA; f Center for Disease Control and Prevention/National Institute for Occupational Safety and Health, Atlanta, GA, USA; g Medical Research Council Social, Genetic and Developmental Psychiatry Centre, Institute of Psychiatry, Kings College, London, UK; h Center for Research in Occupational Health (CiSAL), Universitat Pompeu Fabra, Barcelona, Spain; i Epidemiology and Preventive Medicine Research Center, University of Insubria, Varese, Italy; j Department of Social Medicine, Medical School, University of Crete, Heraklion, Greece; k Department of Public Health, University of Tartu, Estonia; l Department of Environmental Health, Faculty of Health Sciences, American University of Beirut, Beirut, Lebanon; m Department of Occupational Health, Faculty of Health, Shahroud University of Medical Sciences, Shahroud, Iran; n Department of Community Health Sciences, Aga Khan University, Karachi, Pakistan; o Department of Medical Education and Health Sciences, Faculty of Medical Sciences, University of Sri Jayewardenepura, Gangodawila, Nugegoda, Sri Lanka; p Clinical Research Centre for Occupational Musculoskeletal Disorders, Kanto Rosai Hospital, Kawasaki, Japan; q National Institute for Occupational Health, National Health Laboratory Service, Johannesburg, South Africa; r Faculty of Health Sciences, University of Witwatersrand, Johannesburg, South Africa; s Department of Epidemiology and Preventive Medicine, School of Public Health and Preventive Medicine, Monash University, Melbourne, VIC, Australia; t Department of Preventive and Social Medicine, University of Otago, Dunedin, New Zealand; u School of Nursing of Ribeirão Preto, University of São Paulo, São Paulo, Brazil; v Federal University of Paraná, Curitiba-PR, Brazil; w Institute for Studies on Toxic Substances (IRET), National University of Costa Rica, Heredia, Costa Rica; x CIBER of Epidemiology and Public Health, Barcelona, Spain; y Occupational Health Service, Parc de Salut MAR, Barcelona, Spain; z Department of Clinical Sciences and Community Health, Università degli Studi di Milano, Milan, Italy; aa Fondazione Ca’ Granda Ospedale Maggiore Policlinico, Milan, Italy; ab Department of Psychiatry, Medical School, University of Crete, Heraklion, Greece; ac Centre for Research in Environmental Epidemiology (CREAL), Barcelona, Spain; ad IMIM (Hospital del Mar Research Institute), Barcelona, Spain; ae National School of Public Health, Athens, Greece; af North Estonia Medical Centre, Tallinn, Estonia; ag Põlva Hospital, Põlva, Estonia; ah Klinikum Leverkusen, Leverkusen, Germany; ai Department of Physiology, Faculty of Medical Sciences, University of Sri Jayewardenepura, Gangodawila, Nugegoda, Sri Lanka; aj Section of Epidemiology and Biostatistics, School of Population Health, Faculty of Medical and Health Sciences, University of Auckland, Auckland, New Zealand; ak Department of Epidemiology, School of Public Health, University of Alabama at Birmingham, Birmingham, AL, USA; al Faculty of Medicine,University of Kalaniya, Kelaniya, Sri Lanka; am Department of Joint Disease Research, University of Tokyo, Tokyo, Japan; an Centre for Occupational and Environmental Health, Department of Social and Preventive Medicine, Faculty of Medicine, University of Malaya, Kuala Lumpur, Malaysia; ao Injury Prevention Research Unit, Department of Preventive and Social Medicine, University of Otago, Dunedin, New Zealand

**Keywords:** Pain, Multisite, Widespread, Definition, Risk factors

## Abstract

To explore definitions for multisite pain, and compare associations with risk factors for different patterns of musculoskeletal pain, we analysed cross-sectional data from the Cultural and Psychosocial Influences on Disability (CUPID) study. The study sample comprised 12,410 adults aged 20–59 years from 47 occupational groups in 18 countries. A standardised questionnaire was used to collect information about pain in the past month at each of 10 anatomical sites, and about potential risk factors. Associations with pain outcomes were assessed by Poisson regression, and characterised by prevalence rate ratios (PRRs). Extensive pain, affecting 6–10 anatomical sites, was reported much more frequently than would be expected if the occurrence of pain at each site were independent (674 participants vs 41.9 expected). In comparison with pain involving only 1–3 sites, it showed much stronger associations (relative to no pain) with risk factors such as female sex (PRR 1.6 vs 1.1), older age (PRR 2.6 vs 1.1), somatising tendency (PRR 4.6 vs 1.3), and exposure to multiple physically stressing occupational activities (PRR 5.0 vs 1.4). After adjustment for number of sites with pain, these risk factors showed no additional association with a distribution of pain that was widespread according to the frequently used American College of Rheumatology criteria. Our analysis supports the classification of pain at multiple anatomical sites simply by the number of sites affected, and suggests that extensive pain differs importantly in its associations with risk factors from pain that is limited to only a small number of anatomical sites.

## Summary

In a large cross-sectional survey, pain affecting 6–10 anatomical sites showed substantially different associations with risk factors from pain limited to 1–3 sites.

## 1 Introduction

Musculoskeletal pain often occurs simultaneously at more than one anatomical site, and there is a case that pain with a distribution that is unusually widespread should be viewed as a separate clinical entity, distinct from more localised pain. Various criteria have been advanced by which to define widespread pain [11], [16], [27], [28], [29]. In particular, the American College of Rheumatology (ACR) has proposed that pain should be classed as widespread if it occurs axially, in at least one upper limb, and also in a contralateral lower limb [28], [29].

Others have argued that pain occurs in a continuum of severity characterised by the number of sites that are painful [12], implying that there is no fundamental distinction between widespread pain and pain that is more localised. In support of this view, longitudinal studies have demonstrated that over time, transition between diagnoses of localised and widespread pain (in either direction) is quite common [9], [13], [15], [21].

In the absence of a clear gold standard related to pathogenesis, the validity of diagnostic criteria depends on their ability to distinguish usefully a group of people with illness that has distinctive risk factors, prognosis, or response to treatment [2]. Epidemiological studies have established various risk factors for chronic widespread pain, including female sex [1], [6], [14], [24], older age [1], [6], tendency to somatise [6], [7], [10], [11], [15], [18], [22], [23], and depression or mental distress [6], [11], [12], [16], [17]. In addition, elevated risks have been found for various physically stressing occupational activities [18]. However, it is unclear whether associations with these risk factors differ importantly from those for more limited musculoskeletal pain.

Furthermore, if there is value in distinguishing widespread from other categories of pain, then clarification is required regarding its optimal definition. The ACR criteria have face validity, and were met by almost all of a series of patients with a clinical diagnosis of fibromyalgia, as compared with only 69% of a control group who suffered from other disorders that might be confused with fibromyalgia [29]. However, clinical diagnosis of fibromyalgia cannot be considered a robust gold standard, and it may be that other case definitions would perform better. An alternative approach might be to distinguish those patterns of multisite pain, which are found with higher frequency than would be expected if the occurrence of pain at each individual anatomical site were statistically independent.

To explore possible definitions for multisite pain, and compare associations with risk factors for different patterns of musculoskeletal pain, we analysed baseline data from the Cultural and Psychosocial Influences on Disability (CUPID) study [3].

## 2 Methods

The CUPID study sample comprised workers aged 20–59 years from 47 occupational groups (office workers, nurses, and “other workers”) in 18 countries (Table 1). During 2006–2011, participants completed a standardised questionnaire about musculoskeletal pain, associated disability, and possible risk factors, either at interview (25 groups), by self-administration (18 groups), or a combination of interview and self-administration (4 groups). Response rates among those invited to take part were mostly higher than 80% (33 groups), but were lower than 50% in 5 groups. For logistic reasons, data collection was earlier in some countries than in others.

**Table 1:** Occupational groups included in the CUPID study.

Country	Occupational group	Response rate (%)	Number of subjects in CUPID study sample	Number of subjects in current analysis
Brazil (BR)	Nurses	96	185	185
	Office workers	97	281	281
	Other workers (sugar cane cutters)	61	93	93
Ecuador (EC)	Nurses	99	219	219
	Office workers	100	243	243
	Other workers (flower plantation)	99	227	227
Colombia (CO)	Office workers	89	92	92
Costa Rica (CR)	Nurses	91	220	220
	Office workers	91	223	223
	Other workers (telephone call centre)	94	205	205
Nicaragua (NI)	Nurses	100	282	282
	Office workers	100	285	285
	Other workers (machine operators)	100	197	197
UK (UK)	Nurses	42	257	257
	Office workers	45	380	380
	Other workers (mail sorters)	28	386	386
Spain (SP)	Nurses	96	667	666
	Office workers	98	438	437
Italy (IT)	Nurses	76	536	535
	Other workers (assembly line)	52	139	137
Greece (GR)	Nurses	93	224	224
	Office workers	99	199	199
	Other workers (postal clerks)	91	140	140
Estonia (EE)	Nurses	48	371	371
	Office workers	53	202	202
Lebanon (LB)	Nurses	96	184	184
	Office workers	86	172	172
	Other workers (food production)	98	137	137
Iran (IR)	Nurses	94	246	246
	Office workers	88	182	182
Pakistan (PK)	Nurses	94	187	187
	Office workers	100	180	180
	Other workers (mail sorters)	96	222	222
Sri Lanka (LK)	Nurses	95	236	236
	Office workers	63	152	152
	Other workers-1 (mail sorters)	100	250	250
	Other workers-2 (sewing machinists)	86	151	151
Japan (JP)	Nurses	76	592	590
	Office workers	81	310	310
	Other workers-1 (transportation operatives)	86	1018	1010
	Other workers-2 (sales workers)	98	355	354
South Africa (SA)	Nurses	90	247	247
	Office workers	83	229	229
Australia (AU)	Nurses	39	250	250
New Zealand (NZ)	Nurses	70	177	177
	Office workers	52	145	145
	Other workers (mail sorters)	50	113	113

CUPID, Cultural and Psychosocial Influences on Disability.

The questionnaire was originally drafted in English, and then translated into local languages where necessary. The accuracy of translation was checked by independent back-translation, and amendments were made if needed. Among other things, the questionnaire asked whether during the past month, pain had been present for a day or longer in each of 6 anatomical regions (low back, neck, shoulder, elbow, wrist/hand, and knee) depicted in diagrams, and for the limb regions, whether the pain had been on the right, left, or both sides. It also asked about sex, age, age at which full-time education was completed, smoking habits, somatising tendency, mental health, physical activities at work, psychosocial aspects of work, and fear-avoidance beliefs about musculoskeletal pain.

Somatising tendency was assessed using questions from the Brief Symptom Inventory [8], and graded according to the number of common somatic symptoms from a total of 5 (faintness or dizziness, pains in the heart or chest, nausea or upset stomach, trouble getting breath, hot or cold spells) that had been at least moderately distressing in the past week. Questions about mental health came from the relevant domain of the Short Form-36 questionnaire [26], and scores were classified to approximate thirds of the distribution in the full study sample (denoted good, intermediate, and poor).

Exposure to physical loading at work was scored according to how many of 5 activities (lifting weights of 25 kg or more by hand; working for longer than 1 hour in total with the hands above shoulder height; repeated bending and straightening of the elbow for longer than 1 hour in total; use of a computer keyboard or other repeated movements of the wrist or fingers for longer than 4 hours in total; and kneeling or squatting for longer than 1 hour in total) were reported in an average working day. Time pressure at work was considered to be present if a participant reported either a target number of articles or tasks to be finished in the working day, or working under pressure to complete tasks by a fixed time. Lack of support at work was deemed to occur if help with difficulties was seldom or never provided by colleagues or a supervisor/manager. Job dissatisfaction was classed as present if overall, the participant felt dissatisfied or very dissatisfied with their employment. Lack of control was considered to occur if there was seldom or never choice in all of: a) how work was done, b) what was done at work, and c) work timetable and breaks. Job insecurity was taken as present if the participant felt that the tenure of their employment would be “rather unsafe” or “very unsafe” if they were off work for 3 months with significant illness.

Questions concerning fear-avoidance beliefs were adapted from the Fear Avoidance Beliefs Questionnaire [25]. Participants were deemed to have adverse beliefs about the work-relatedness of musculoskeletal pain if they completely agreed that either low-back pain or arm pain is commonly caused by people’s work; about physical activity if either for someone with low-back pain or for someone with arm pain, they completely agreed both that physical activity should be avoided as it might cause harm, and that rest was needed to get better; and about prognosis if either for low-back pain or arm pain, they completely agreed that neglecting such problems can cause permanent health problems, and completely disagreed that such problems usually get better within 3 months.

Further details of the methods of the CUPID study sample and methods of data collection have been reported elsewhere [3].

Statistical analysis was carried out with Stata 12.1 software (StataCorp LP, College Station, TX, USA). We first calculated the prevalence of pain in the past month at each of 10 anatomical sites (low back, neck, right shoulder, left shoulder, right elbow, left elbow, right wrist/hand, left wrist/hand, right knee, and left knee) and summarised the associations between pain at pairs of sites by odds ratios (ORs).

Next we classified subjects according to the number of anatomical sites (from 0 to 10) that they reported as having been painful in the past month, and compared the observed frequencies with the numbers that would have been expected given the overall prevalence of pain at each site by sex and age, and assuming that the occurrence of pain at any 2 sites was independent. For example, if within a specified sex and age group, the prevalence of pain in the 10 sites was P_1_, P_2_, … P_10_, then in that group, the expected prevalence of no pain at any of the 10 sites would be $\prod_{i=1}^{10}(1-P_{i})$ and that of pain at all 10 sites $\prod_{i=1}^{10}(P_{i})$. Ratios of observed to expected counts (O/E) were calculated for the full study sample, and broken down according to whether or not the distribution of pain was widespread (ie, it was reported in each of the trunk, upper limb and lower limb, and also on both sides of the body). This analysis was used to define “limited pain” involving a small number of sites with O/E < 1, and “extensive pain” involving a large number of sites with O/E clearly > 1.

We then explored personal risk factors for musculoskeletal pain affecting different numbers of anatomical sites. We used Generalised Linear Latent and Mixed Models (GLLAMM) to fit 2-level, random intercept Poisson regression models with robust standard errors, in which individuals were clustered by occupational group. Associations were summarised by prevalence rate ratios (PRRs) with associated 95% confidence intervals (95% CIs). To check the robustness of the findings, we repeated the analyses, using 2-level, random intercept logistic regression models.

To check whether a pattern of pain that was widespread (ie, in the trunk, on both sides of the body, and in both an upper and lower limb) showed additional association with risk factors after the number of sites with pain had been taken into account, we carried out a Poisson regression analysis with widespread pain as the outcome, adjusting for the number of sites with pain (treated as dummy variables).

Next, we constructed single-level Poisson regression models with limited and extensive pain as the outcome variables, and incorporating occupational group as an independent variable while adjusting for all of the personal risk factors examined previously. For this purpose, office workers in the UK were taken as the reference group for risk estimates, and the PRRs for limited and extensive pain (relative to no pain) were compared across the 47 occupational groups to see whether they correlated.

Finally, we carried out sensitivity analyses in which we repeated the Poisson regression analyses: a) excluding the 5 occupational groups with response rate < 50%; and b) adjusting also for the method by which the questionnaire was answered (interview or self-administration).

## 3 Results

The total CUPID study sample comprised 12,426 participants [3], but 16 were excluded from the current analysis because of incomplete information about the occurrence of pain at some anatomical sites. Among the remaining 12,410, the number by occupational group ranged from 92 to 1010 (Table 1).

Table 2 shows the 1-month prevalence of pain at each of the 10 anatomical sites, and the associations between pain at pairs of sites, summarised by ORs. Pain was reported most frequently in the low back (35.7%) and neck (31.0%), and least often in the right elbow (6.6%) and left elbow (4.3%). Participants who had pain at one anatomical site were more likely than those who did not, to have pain at other given sites (ORs ⩾2.5 for all pairs of sites). However, the strongest associations were for pain at corresponding sites bilaterally (ORs 23.0 for right and left knee, 21.5 for right and left elbow, 18.7 for right and left wrist/hand, and 10.8 for right and left shoulder). Higher ORs (5.0 to 10.2) were also observed for pain at adjacent anatomical sites in the upper limb (neck with shoulder, shoulder with ipsilateral elbow, and elbow with ipsilateral wrist/hand).

**Table 2:** Prevalence of pain in the past month at 10 anatomical sites and associations between pain at pairs of sites.

Anatomical site	Prevalence (%) of pain in past month	Odds ratios (with 95% confidence intervals) for associations with pain at other anatomical sites^a^								
		Low back	Neck	Right shoulder	Left shoulder	Right elbow	Left elbow	Right wrist/hand	Left wrist/hand	Right knee
Low back	35.7									
Neck	31.0	3.8								
		(3.5–4.1)								
Right shoulder	18.7	3.0	5.2							
		(2.8–3.3)	(4.7–5.7)							
Left shoulder	14.2	3.0	5.0	10.8						
		(2.7–3.3)	(4.4–5.5)	(9.6–12.1)						
Right elbow	6.6	2.7	3.2	5.5	2.7					
		(2.3–3.1)	(2.8–3.7)	(4.7–6.4)	(2.3–3.1)					
Left elbow	4.3	3.4	3.1	2.8	6.1	21.5				
		(2.8–4.1)	(2.6–3.8)	(2.3–3.4)	(5.0–7.3)	(17.7–26.1)				
Right wrist/hand	15.9	3.0	3.1	3.8	2.5	7.1	4.2			
		(2.7–3.4)	(2.8–3.5)	(3.4–4.2)	(2.3–2.9)	(6.1–8.2)	(3.5–5.0)			
Left wrist/hand	10.1	2.9	3.0	2.6	4.1	3.8	10.2	18.7		
		(2.6–3.3)	(2.7–3.4)	(2.3–3.0)	(3.6–4.7)	(3.2–4.5)	(8.4–12.3)	(16.3–21.4)		
Right knee	15.0	3.3	2.6	2.8	2.5	4.0	3.6	3.2	2.8	
		(3.0–3.6)	(2.3–2.9)	(2.5–3.2)	(2.2–2.8)	(3.5–4.7)	(3.0–4.3)	(2.8–3.6)	(2.4–3.2)	
Left knee	14.1	3.3	2.6	2.6	2.7	3.3	3.9	2.8	3.4	23.0
		(2.9–3.6)	(2.3–2.9)	(2.3–2.9)	(2.4–3.0)	(2.8–3.9)	(3.3–4.7)	(2.5–3.2)	(3.0–3.8)	(20.4–26.1)

a Odds ratios are adjusted for sex and age (in 10-year bands).

Table 3 shows how often pain was reported at different numbers of anatomical sites, and compares the observed counts with the frequencies that would have been expected based on the overall prevalence of pain at each anatomical site by sex and age, and assuming that the occurrence of pain at each anatomical site was statistically independent. Results are presented for all distributions of pain, and also broken down according to whether or not the distribution of pain was widespread. As would be expected from the associations in Table 2, overall there were more participants than expected with no pain at any of the 10 sites (O/E = 2.16). In contrast, there were fewer than expected with pain at just 1, 2, or 3 sites (O/E = 0.667, 0.539, and 0.695, respectively). However, the frequency with which 6 or more sites were reported as painful was well above expectation (674 participants vs 41.9 expected), the O/E ratio increasing progressively from 8.87 for 6 sites to 86,900 for all 10 sites (44 participants). Most people with pain at 6–10 sites met the criteria for widespread pain (81%), whereas among those with pain at 4–5 sites, only 33% had widespread pain. When the analysis was broken down according to the distribution of pain, the O/E ratio for pain at any given number of sites was consistently greater when the pain was not classed as widespread. Based on this analysis, we defined pain at 1–3 anatomical sites as “limited” and pain at 6–10 sites as “extensive.”

**Table 3:** Observed and expected distributions of participants according to number of anatomical sites with pain in past month.

Number of anatomical sites with pain in past month	Pain not widespread^a^			Pain widespread^a^			All distributions of pain		
	Observed number of subjects	Expected number of subjects^b^	Ratio of observed to expected	Observed number of subjects	Expected number of subjects^b^	Ratio of observed to expected	Observed number of subjects	Expected number of subjects^b^	Ratio of observed to expected
0	4638	2150	2.16	0	0	-	4638	2150	2.16
1	2660	3990	0.667	0	0	-	2660	3990	0.667
2	1866	3460	0.539	0	0	-	1866	3460	0.539
3	1257	1630	0.773	47	250	0.188	1304	1880	0.695
4	647	429	1.51	171	274	0.624	818	703	1.16
5	202	65.3	3.09	248	124	2.00	450	189	2.38
6	103	6.06	17.0	221	30.5	7.25	324	36.5	8.87
7	17	0.321	52.9	134	4.59	29.2	151	4.91	30.7
8	10	0.008	1230	98	0.426	230	108	0.434	249
9	0	0	-	47	0.022	2100	47	0.022	2100
10	0	0	-	44	0.001	86900	44	0.001	86900

a Pain was classed as widespread if it occurred in the trunk (low back or neck), on both sides of the body, and in both an upper and lower limb.

b Expected numbers were calculated from the prevalence of pain at each anatomical site by sex and 10-year age band, with the assumption that the occurrence of pain at each site was statistically independent (see text).

Table 4 summarises the associations of pain at different numbers of anatomical sites with personal risk factors. For each pain outcome, risk estimates are relative to people with no pain, and were derived from a single regression model that included all of the variables for which results are presented. Limited pain (1–3 sites) showed only modest associations with the risk factors examined, the strongest being for report of 5 physically loading activities (PRR 1.4, 95% CI 1.2–1.6) and somatising tendency (PRR 1.3, 95% CI 1.2–1.4) for report of 2 or more distressing somatic symptoms. However, for many risk factors, the PRRs for extensive pain (6–10 sites) were substantially higher. These included female sex (1.6), older age (2.6), report of 2 or more distressing somatic symptoms (4.6), and exposure to multiple physically loading activities (up to 5.0). Corresponding risk estimates for pain at 4–5 sites were intermediate between those for limited and extensive pain. When the analysis was repeated using logistic regression (ie, with characterisation of associations by ORs rather than PRRs), the pattern of results was similar (data not shown).

**Table 4:** Associations with risk factors according to number of anatomical sites with pain.

Risk factor	Number of anatomical sites with pain									
	0	1–3			4–5			6–10		
	n	n	PRR^a^	(95% CI)	n	PRR^a^	(95% CI)	n	PRR^a^	(95% CI)
Sex										
Male	2059	1861	1		306	1		116	1	
Female	2579	3969	1.1	(1.0–1.2)	962	1.4	(1.2–1.7)	558	1.6	(1.2–2.1)
Age (years)										
20–29	1327	1422	1		217	1		93	1	
30–39	1554	1902	1.1	(1.0–1.1)	355	1.3	(1.1–1.5)	156	1.3	(1.0–1.7)
40–49	1136	1649	1.1	(1.0–1.2)	407	1.7	(1.4–2.0)	253	2.2	(1.6–2.9)
50–59	621	857	1.1	(1.0–1.2)	289	2.1	(1.7–2.6)	172	2.6	(1.9–3.7)
Age finished full-time education (years)										
⩾20	2745	3410	1		720	1		365	1	
17–19	1285	1564	1.0	(1.0–1.1)	345	1.1	(1.0–1.2)	176	1.1	(1.0–1.2)
14–16	440	629	1.1	(1.0–1.1)	125	1.0	(0.8–1.2)	78	1.0	(0.9–1.3)
<14	142	201	1.0	(0.9–1.1)	72	1.0	(0.7–1.3)	54	1.0	(0.8–1.4)
Unknown	26	26	1.0	(0.7–1.3)	6	0.8	(0.4–1.4)	1	0.3	(0.1–0.7)
Smoking status										
Never smoked	3044	3558	1		785	1		458	1	
Ex-smoker	586	918	1.1	(1.1–1.2)	190	1.2	(1.1–1.4)	88	1.1	(0.9–1.3)
Current smoker	985	1343	1.1	(1.0–1.1)	291	1.2	(1.1–1.4)	125	1.1	(0.9–1.3)
Missing	23	11	0.7	(0.4–1.1)	2	0.4	(0.2–1.2)	3	0.8	(0.3–2.0)
Number of distressing somatic symptoms in past week										
0	3429	3327	1		489	1		153	1	
1	755	1363	1.2	(1.1–1.2)	346	1.9	(1.7–2.1)	143	2.6	(2.0–3.3)
2+	408	1086	1.3	(1.2–1.4)	417	2.4	(2.1–2.8)	369	4.6	(3.5–6.1)
Missing	46	54	1.1	(0.9–1.2)	16	1.6	(1.1–2.3)	9	2.7	(1.4–5.0)
Mental health										
Good	2075	2092	1		360	1		172	1	
Intermediate	1348	1838	1.1	(1.1–1.2)	400	1.4	(1.2–1.5)	172	1.1	(0.9–1.4)
Poor	1178	1870	1.2	(1.2–1.3)	502	1.6	(1.4–1.9)	327	1.6	(1.3–1.9)
Missing	37	30	0.9	(0.7–1.3)	6	0.8	(0.4–1.7)	3	0.7	(0.2–1.9)
Number of physically loading activities										
0	470	356	1		41	1		10	1	
1	972	1023	1.1	(1.0–1.2)	145	1.3	(1.0–1.8)	56	1.8	(1.0–3.3)
2	1361	1753	1.2	(1.1–1.3)	382	1.9	(1.4–2.6)	214	3.2	(1.9–5.3)
3	1105	1443	1.2	(1.1–1.4)	347	2.2	(1.6–3.0)	171	3.2	(1.9–5.3)
4	523	851	1.3	(1.2–1.5)	240	2.6	(1.9–3.6)	135	4.0	(2.2–7.1)
5	207	404	1.4	(1.2–1.6)	113	2.8	(2.0–4.0)	88	5.0	(2.8–9.2)
Psychosocial aspects of work										
Work ⩾50 hours per week	1300	1102	1.0	(0.9-1.0)	183	0.9	(0.8–1.1)	75	0.9	(0.6–1.1)
Time pressure at work	3335	4436	1.1	(1.0–1.1)	1015	1.2	(1.1–1.4)	547	1.2	(1.0–1.5)
Lack of support at work	847	1506	1.1	(1.0–1.1)	386	1.2	(1.0–1.3)	271	1.3	(1.1–1.5)
Job dissatisfaction	971	1137	1.0	(0.9–1.1)	271	1.1	(0.9–1.3)	154	1.0	(0.8–1.2)
Lack of job control	989	1162	1.0	(0.9–1.0)	314	1.1	(1.0–1.2)	199	1.1	(1.0–1.3)
Job insecurity	1473	1775	1.0	(1.0–1.1)	388	1.0	(0.9–1.1)	274	1.2	(1.0–1.4)
Adverse beliefs about musculoskeletal pain										
Work-relatedness	1521	2326	1.1	(1.1–1.1)	644	1.5	(1.3–1.6)	378	1.5	(1.2–1.7)
Physical activity	1042	1165	0.9	(0.9–1.0)	239	0.7	(0.6–0.9)	131	0.8	(0.7–1.0)
Prognosis	558	1057	1.1	(1.1–1.2)	285	1.3	(1.2–1.5)	178	1.3	(1.2–1.5)

PRR, prevalence rate ratio; CI, confidence interval.

a Mutually adjusted prevalence rate ratios in comparison with no pain at any anatomical site.

Table 5 shows the relation of widespread pain to sex, age, somatising tendency, and number of physically loading occupational activities after adjustment for the number of sites with pain. This analysis was restricted to participants with pain at 3–8 anatomical sites since no-one with pain at fewer than 3 sites could have widespread pain, and pain met the criteria for being widespread in all who had pain at 9 or 10 sites. After adjustment for number of sites with pain, and also for all of the other risk factors in the Table, there was no indication of any additional association with widespread pain.

**Table 5:** Associations of widespread pain with risk factors.

Risk factor	Number of subjects^a^	Number (%) of subjects with widespread pain		PRR^b^	(95% CI)
Sex					
Male	741	207	(27.9)	1	
Female	2414	712	(29.5)	0.9	(0.8–1.0)
Age (years)					
20–29	577	147	(25.5)	1	
30–39	901	227	(25.2)	0.9	(0.8–1.1)
40–49	1025	316	(30.8)	1.0	(0.8–1.1)
50-59	652	229	(35.1)	1.0	(0.9–1.2)

Number of distressing somatic symptoms in past week					
0	1276	281	(22.0)	1	
1	802	218	(27.2)	1.0	(0.9–1.1)
2+	1036	410	(39.6)	1.1	(0.9–1.2)
Missing	41	10	(24.4)	1.0	(0.7–1.4)

Number of physically loading activities					
0	110	22	(20.0)	1	
1	420	106	(25.2)	1.1	(0.8–1.4)
2	938	285	(30.4)	1.0	(0.7–1.3)
3	823	221	(26.9)	0.9	(0.7–1.3)
4	578	195	(33.7)	1.0	(0.7–1.4)
5	286	90	(31.5)	0.9	(0.6–1.3)

PRR, prevalence rate ratio; CI, confidence interval.

a Analysis was restricted to subjects with pain at 3–8 anatomical sites (for explanation see text).

b Risk estimates are for widespread pain relative to pain that was not widespread according to American College of Rheumatology criteria, and are adjusted for all risk factors in the Table, and also for number of sites with pain (3, 4, 5, 6, 7, or 8).

**Fig. 1:**
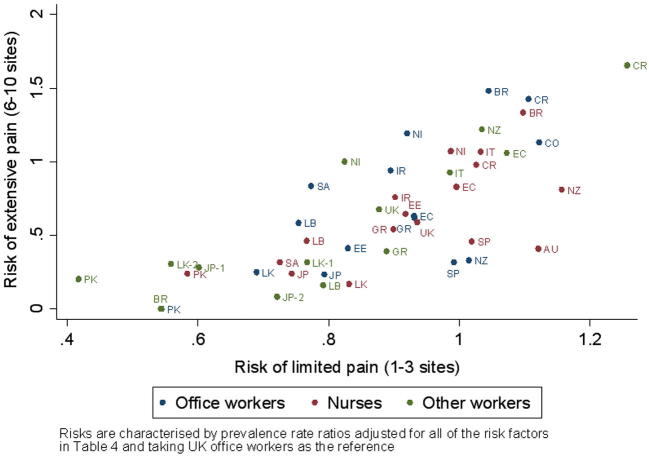
Risk of extensive and limited pain by occupational group.

Fig. 1 compares PRRs for limited and extensive pain in the 47 occupational groups that made up the study sample. These were derived from 2 Poisson regression models with adjustment for all of the personal risk factors examined in Table 4, and taking the risk in office workers in UK as the reference. After adjustment for personal risk factors, there was substantial variation in both outcomes, the PRR for limited pain varying from 0.4 (95% CI 0.3–0.5) in Pakistani manual workers to 1.3 (95% CI 1.1–1.4) in manual workers in Costa Rica, and that of extensive pain from 0.0 (95% CI 0.0–0.0) in Pakistani office workers and Brazilian sugar cane cutters to 1.7 (95% CI 0.9–2.9) in manual workers in Costa Rica (the corresponding prevalence rates in these 5 occupational groups were 25%, 82%, 0%, 0%, and 56%, respectively). PRRs for the 2 categories of pain were correlated (Spearman correlation coefficient = 0.76), occupational groups from Asian countries tending to have low rates for both pain outcomes, and most occupational groups from Central and South America lying at the upper end of the range. However, within the overall correlation, there was notable variation. For example, among the 11 occupational groups with a PRR of 0.95-1.05 for limited pain, PRRs for extensive pain ranged from 0.3 (95% CI 0.1–0.7) in Spanish office workers to 1.5 (95% CI 0.8–2.6) in Brazilian office workers.

## 4 Discussion

In this analysis, associations between pain at pairs of anatomical sites were strongest for corresponding sites on the right and left of the body, and for adjacent sites in the neck and upper limb. Nevertheless, the occurrence of pain at any given number of sites was more exceptional (as compared with what would have been expected if pain at each site were independent) when it did not meet the ACR criteria for being widespread. Extensive pain (involving 6–10 sites) showed much stronger associations with physical and psychosocial risk factors than limited pain (involving only 1–3 sites), but after adjustment for the number of sites with pain, there were no additional associations with a widespread distribution of pain. This suggests that in studies of causation, it might be better for classification of pain at multiple sites to be based simply on the number of sites affected, regardless of their anatomical distribution. Furthermore, although relative risks of extensive and limited pain by occupational group were correlated, there were notable deviations from the overall pattern. This points to other determinants that differ for the 2 categories of pain, and adds to the case for treating them as distinct outcomes.

Our study had the advantage that it used standardised questions to collect data on pain and risk factors from a large sample of participants. Response rates were generally high, and exclusion of occupational groups in which response was lower did not materially alter the results. Nor were findings importantly different when adjusted for whether the questionnaire was self-administered or completed at interview.

Care was taken in translation of the questionnaire, but it remains possible that the concept of pain was understood differently in different languages. This would be expected to affect limited and extensive pain similarly, and may have contributed to some of the variation between occupational groups that remained after adjustment for established risk factors. However, major differences in risk were apparent even between occupational groups questioned in the same language (eg, groups in Costa Rica and Colombia as compared with Spain, and nurses and office workers in Brazil as compared with sugar cane cutters in the same country). Furthermore, as we have reported elsewhere, similar differences were observed also in the prevalence of pain that was reported as disabling for everyday activities, or to have caused sickness absence [4], [5], outcome variables which are less likely to be biased by nuances of translation.

Because the study was cross-sectional, we cannot exclude the possibility that some of the observed associations with risk factors reflected reverse causation. For example, it would not be surprising if musculoskeletal pain lowered people’s mood, especially if extensive. To the extent that this occurred, it would have inflated risk estimates for the risk factors concerned, and possibly led to over-adjustment when comparing risk between occupational groups. However, it would not be expected to cause spurious variation between occupational groups.

Bias may also have occurred because performance of certain physical activities at work made participants more aware of, and more likely to report, pain. However, this could not explain the large differences in pain prevalence that were observed between groups of office workers in different countries, whose activities were generally similar [3]. On the other hand, between-group variation may have been over-estimated to some extent if there were non-differential errors in the assessment and classification of factors of adjustment. Because of limited resources, it was not possible to assess occupational activities by direct observation.

That pain at one anatomical site was often associated with pain at an adjacent site, or at the same site on the other side of the body, was unsurprising. The relationship may be explained in part by shared risk factors, both physical and psychosocial. In addition, pain that is diffuse or radiating may not localise to a single site as defined in our questionnaire. Given this pattern of association, it might be expected that widespread pain, which involved the trunk, upper limb and lower limb, and also both sides of the body, would be particularly unusual. However, we found that the extent to which a distribution of pain was unusual depended more on the number of sites affected than on whether it met the definition for being widespread. Moreover, by comparing the observed numbers of affected anatomical sites with the numbers that would have been expected if pain at each site were independent, we were able to define a threshold number of painful sites (n = 6), at or above which the distribution of symptoms was clearly exceptional.

When we applied this definition of extensive pain, we found much stronger associations with sex, age, somatising tendency, and exposure to physically loading activities at work, than for limited pain. In contrast, the differential for other risk factors, such as smoking, poor mental health, and psychosocial aspects of work, was much smaller. Risk estimates for pain affecting 4–5 sites, which was less remarkable (1268 participants vs 892 expected), were intermediate. Prevalence rate ratios are constrained insofar as prevalence cannot exceed 100%, and this might prevent PRRs for more common health outcomes, such as limited pain, attaining such high values as those for rarer outcomes such as extensive pain. However, when we repeated our analysis using logistic regression (with odds ratio as the measure of association) instead of Poisson regression, the pattern was similar, demonstrating that this was not the explanation for the higher risk estimates for extensive pain. Furthermore, there was no indication of additional associations with pain being widespread, once the number of sites with pain had been taken into account (Table 5).

While other studies have explored risk factors for widespread or multisite pain, with findings broadly similar to ours [1], [6], [7], [10], [11], [12], [14], [15], [16], [17], [18], [19], [22], [23], [24], few investigations have examined associations with pain confined to only 1 or 2 anatomical sites. A study in Norway found a higher prevalence of pain at 6–10 anatomical sites relative to 1–3 sites in women as compared with men [14], and that among subjects with back pain, widespread pain was more common in women, people of middle age, and those with more than moderate emotional problems [20]. Our findings are consistent with these observations, and suggest that localised musculoskeletal pain that is not accompanied by pain at multiple other anatomical sites may differ in its risk factors from more extensive pain.

After adjustment for established risk factors, there was substantial residual variation between occupational groups in the prevalence of both limited and extensive pain. The broad correlation between risk estimates for the 2 outcomes by occupational group suggests that their variation is driven, at least in part, by the same determinants. However, the correlation was far from exact, with quite marked differences in prevalence of extensive pain between occupational groups with similar prevalence of limited pain. This again points to important differences in the risk factors for limited and extensive pain.

Optimal case definition for multisite pain depends on its practical utility in distinguishing illness with causes, prognosis, or response to treatment that differs importantly from that of pain occurring in other patterns [2]. Our analysis supports the classification of pain at multiple anatomical sites according to the number of sites affected. Although there was a threshold number of painful sites, above which prevalence was clearly higher than would have been expected by chance coincidence, we did not identify a threshold number of affected sites above which associations with risk factors were qualitatively different. Rather, associations became progressively stronger, the larger the number of sites with pain. Nevertheless, risk estimates for several variables were much larger in relation to extensive than limited pain. This suggests that in future research on pain at specific sites such as the back and wrist/hand, there may be value in distinguishing cases with localised pain from those in which pain is more extensive.

## 5 Conflict of interest

None of the authors have any conflicts of interest.
